# Foliar Sprayed Green Zinc Oxide Nanoparticles Mitigate Drought-Induced Oxidative Stress in Tomato

**DOI:** 10.3390/plants10112400

**Published:** 2021-11-07

**Authors:** Manal El-Zohri, Naseem A. Al-Wadaani, Sameera O. Bafeel

**Affiliations:** 1 Department of Biological Sciences, Faculty of Science, King Abdulaziz University, Jeddah 21488, Saudi Arabia; n.botany@hotmail.com (N.A.A.-W.); sbafil@kau.edu.sa (S.O.B.); 2 Department of Botany and Microbiology, Faculty of Science, Assiut University, Assiut 71516, Egypt; 3 Department of Biology, Faculty of Applied Science, Umm Al Qura University, Makkah 21955, Saudi Arabia

**Keywords:** green nanoparticles, water stress, stress indicators, antioxidants, antioxidant enzymes

## Abstract

This study explored the effectiveness of green zinc oxide nanoparticles (ZnO-NPs) foliar spray on tomato growth and oxidative stress relief under drought conditions. Tomato plant subjected to four water regimes (100, 75, 50, and 25% FC), and in the same while seedlings were sprayed with 25, 50, and 100 mg/L green ZnO-NPs. The results showed that tomato growth parameters reduced significantly by increasing drought stress levels, while ZnO-NPs enhanced plant growth under all studied drought levels. Out of three ZnO-NPs concentrations tested, 25 and 50 mg/L ZnO-NPs proved to be the optimum treatments for alleviating drought stress. They increased shoot and root biomass compared to untreated controls. Application of 25 and 50 mg/L ZnO-NPs enhanced shoot dry weight by about 2–2.5-fold, respectively, under severe drought conditions (25%) compared to ZnO-NPs untreated plants. The application of 25 and 50 mg/L green ZnO-NPs decreased the drought-induced oxidative stress as indicated by the reduction in malondialdehyde and hydrogen peroxide concentrations compared to untreated controls. While 100 mg/L ZnO-NPs further increased oxidative stress. The beneficial effects of ZnO-NPs were evident in the plants’ defensive state, in which the concentration of ascorbic acid, free phenols, and the activity of superoxide dismutase, catalase, and ascorbate peroxidase were maintained at higher levels compared to NPs-untreated plants. At severe drought conditions, 25 mg/L ZnO-NPs induced SOD, CAT, and APX activity by about 3.99-, 3.23-, and 2.82-fold of their corresponding controls, respectively. Likewise, at 25% FC, SOD, CAT, and APX activity increased with 50 mg/L ZnO-NPs by about 4.58-, 3.57-, and 3.25-fold consecutively compared with their respective controls. Therefore, foliar use of green ZnO-NPs at lower concentrations might be suggested as an efficient way for enhancing tomato tolerance to drought stress.

## 1. Introduction

Between the abiotic stresses, drought is a major factor affecting the growing and production of crops worldwide [1]. Water deficiency induces a set of physiological and biochemical responses in plants and is one of the most complicated adverse conditions, as it not only depends on the intensity and duration of the stress event but also on the stage of plant growth and morphology [2]. Under conditions of severe drought stress, plant cells undergo oxidative damage as a result of the production of reactive oxygen species (ROS). Among the free radicals of ROS, super oxygen (O_2_^−^), hydroxyl radical (OH), alkoxyl (RO^·^), peroxyl radical (ROO^·^) and non-radicals, hydrogen peroxide (H_2_O_2_), single oxygen (^1^O_2_), and ozone (O_3_) is common [3]. The increase in ROS might adversely damage the cellular organelles, DNA, lipids, and enzymatic configuration and eventually cause cell death [4,5]. Plants have very effective cleaning systems for ROS to protect themselves from destructive oxidative reactions [6]. The antioxidant defense system consists of non-enzymatic antioxidants and some antioxidant enzymes [7].

Recently, nanotechnology has opened new and interesting horizons for plant physiologists in order to improve plant performance under stress conditions [8]. Nanomaterials are materials that have a particle size of 1 to 100 nanometers and incorporate novel physical, chemical, and biological characteristics compared to materials of large size. Researchers believe that the uptake of nanoparticles (NPs) in plants is better than the similar chemicals applied to the plant in bulk shape [9,10,11,12]. An array of physical, chemical, and biological methods has been used to synthesize NPs [13,14,15]. However, the chemical and physical methods have some disadvantages including low production rate, high production cost, and high energy consumption [14,15]. While the biosynthesis of NPs via biological agents such as plant extracts has important features including low cost, short production time, safety, and large-scale production of pure NPs [16]. These green NPs can find a definite place in modern agriculture because they are more compatible, have physiological control, and imitate the natural elicitation of plant defense and antioxidant systems [17]. Therefore, with the increasing progress that has been made through the application of nano-biotechnology tools in the agricultural sector, it is assumed that they will help increase plant growth, productivity, and tolerance of biotic and abiotic stress given that conventional fertilizers can have adverse effects on the environment and possibly on food quality [18].

Micronutrient fertilizers can increase plants’ tolerance to environmental stress such as drought. Zinc is an important micronutrient essential for the optimal growth of plants that carry vital metabolic reactions within plants to promote growing [19]. ZnO-NPs act as fertilizers, growth regulators, pesticides, and herbicides [20]. The foliar application of NPs is considered a more convenient way because the plants can directly absorb as compared to the soil application of chemical fertilizers [21] and consequently decrease soil pollution. The second main advantage of the foliar application of nano-fertilizer is that a very small amount of fertilizer is required as compared to soil application of these materials [22]. Therefore, ZnO-NPs can be considered as an environmentally friendly material that can be used as a green reagent [23]. Recent studies showed that ZnO-NPs application positively regulates plant tolerance to multiple environmental stresses including salinity and water stress [24,25]. Rare information is available regarding the impact of green ZnO-NPs foliar application in vegetables especially tomato under drought stress.

Tomato was chosen as the case study variety because it is a leading protective food generally linked to improved human health through reduced risk of chronic diseases, especially diabetes, cancer, and cardiovascular [26,27]. It is a very important source of important vitamins, minerals, and antioxidants [26]. Despite the important role of tomato in the human diet, its productivity is decreasing worldwide as affected by many abiotic stresses such as heat, salinity, and drought [28,29,30]. Therefore, the aims of the current study were to explore the possible impacts of the foliar application of green ZnO-NPs, biosynthesized using *Coleus forskohlii* leaf extract, on physiological and biochemical attributes including growth, oxidative damage, and antioxidant system of tomato plant under different water levels. Specifically, this research questions whether drought-induced oxidative stress could be alleviated by applying different concentrations of green ZnO-NPs. It was hypothesized that foliar spray of green ZnO-NPs may improve tomato growth by activating the antioxidant system in tomato plants under water-limited environments. To the best of our knowledge, this is the first experiment that demonstrates the protective role of green ZnO-NPs in the absence or presence of drought stress in tomato.

## 2. Results

### 2.1. Growth Parameters

**Figure 1:**
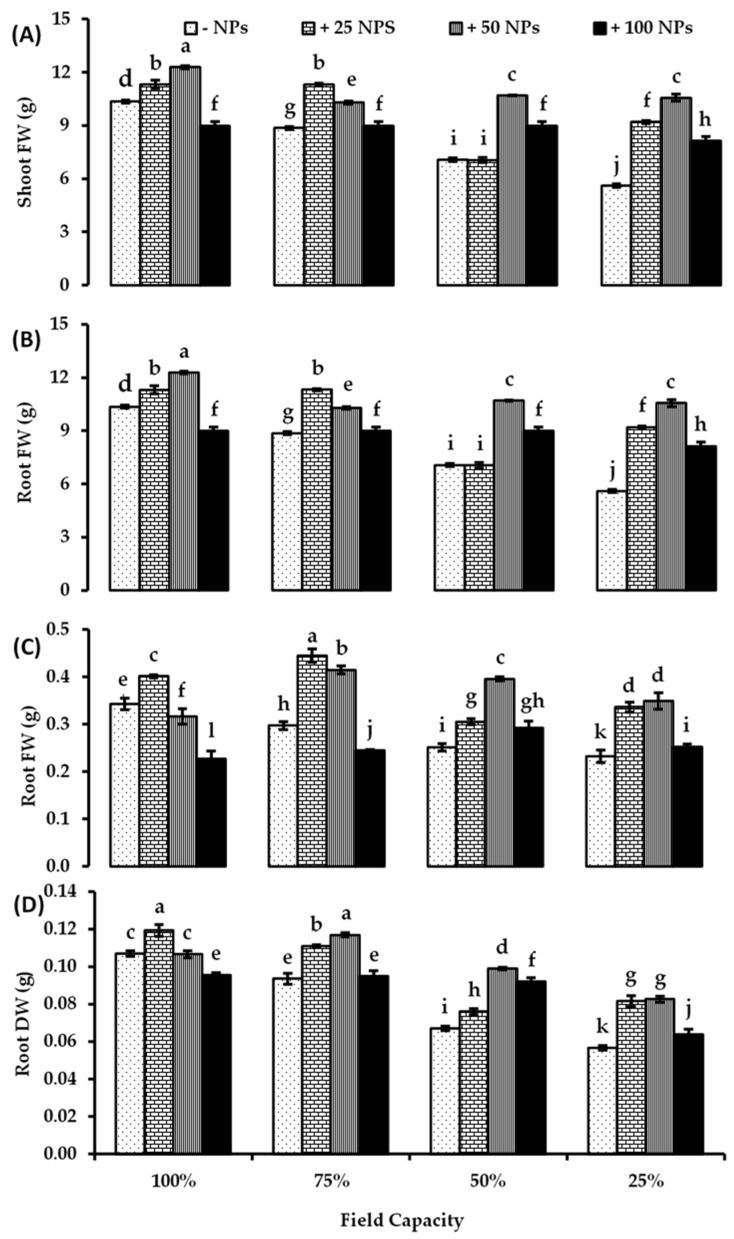
Biomass, (**A**) shoot fresh weight (FW), (**B**) shoot dry weight (DW), (**C**) root fresh weight, and (**D**) root dry weight of tomato plant as affected by foliar spraying with different concentrations of ZnO-NPs under different levels of drought stress. Each point represents a mean value of three replicates (*n* = 3) with vertical bars representing standard error of the mean. Bars with different letters indicate a significant difference (*p* < 0.05) between Zn-O NPs treatments at all studied water levels as determined by two-way ANOVA and Bonferroni multiple comparison test.

Tomato shoot and root biomass decreased gradually by increasing drought stress conditions reaching the lowest values at 25% FC (Figure 1). The variation in growth response of tomato plants in terms of shoot fresh and dry weights is presented in Figure 1A,B. In full watered plants (100% FC), shoot fresh weight (FW) significantly increased (by about 18.67%) when treated with 50 mg/L ZnO-NPs and significantly decreased at higher concentration (100 mg/L ZnO-NPs) compared to untreated control. Under low drought level (75% FC), shoot FW significantly increased when treated with 25 and 50 mg/L ZnO-NPs by about 27.70 and 23.25% higher than their corresponding controls. Moreover, all studied ZnO-NPs concentrations significantly enhance shoot FW under moderate and severe drought conditions (50, 25% FC) compared to their corresponding controls. The most pronounced induction was recorded for 50 mg/L ZnO-NPs treatment, which increased shoot FW by about 50.66 and 88.41% under drought levels of 50 and 25% FC, respectively, compared to their corresponding controls (Figure 1A).

Tomato shoot dry weight (DW) showed similar responses to all studied ZnO-NPs concentrations under the studied water levels. Under full water conditions, shoot DW significantly increased when treated with 25 and 50 mg/L ZnO-NPs compared to untreated control (by about 36%). All studied concentrations of ZnO-NPs markedly increased shoot DW under all drought levels compared to un-treated controls. 25 mg/L ZnO-NPs treatment increased shoot DW by about 35.53, 147.12, 17.133, and 210.09% under all water levels (100, 75, 50, and 25% FC) of un-treated controls, respectively. ZnO-NPs (50 mg/L) increased shoot DW by about 36.28, 114.96, 181.37, and 248.62% at all water regimes systems (100, 75, 50, and 25% FC) respective of their corresponding controls (Figure 1B).

According to the results represented in Figure 1C,D, root fresh and dry weights enhanced variably in response to ZnO-NPs treatments under all studied water levels. Under no drought stress conditions, root FW and DW increased significantly only when treated with 25 mg/L ZnO-NPs. Under 75% FC, root FW and DW were significantly enhanced by about 49.83% and 18.09% when treated with 25 mg/L ZnO, and by about 39.39 and 20.62% when treated with 50 mg/L ZnO-NPs higher than their corresponding controls, respectively (Figure 1C,D). All studied ZnO-NPs treatments significantly enhanced root FW and DW under moderate and severe drought conditions. The highest induction recorded for 50 mg/L ZnO-NPs treatment increased root FW by about 57.77 and 50.43% higher than controls under water levels of 50 and 25% FC, respectively (Figure 1C). In the same context, root DW increased more than 45% higher than controls in response to 50 mg/L ZnO-NPs under water levels of 50 and 25% FC (Figure 1D). Nonetheless, a significant reduction in root FW was recorded due to the treatment with 100 mg/L ZnO under no and mild water stress conditions (100 and 75% FC) (Figure 1C). There were significant effects of drought levels, ZnO-NPs treatments, and their interaction on tomato shoot and root FW and DW based on two-way ANOVA (Table S1). 

### 2.2. Stress Indicators

**Figure 2:**
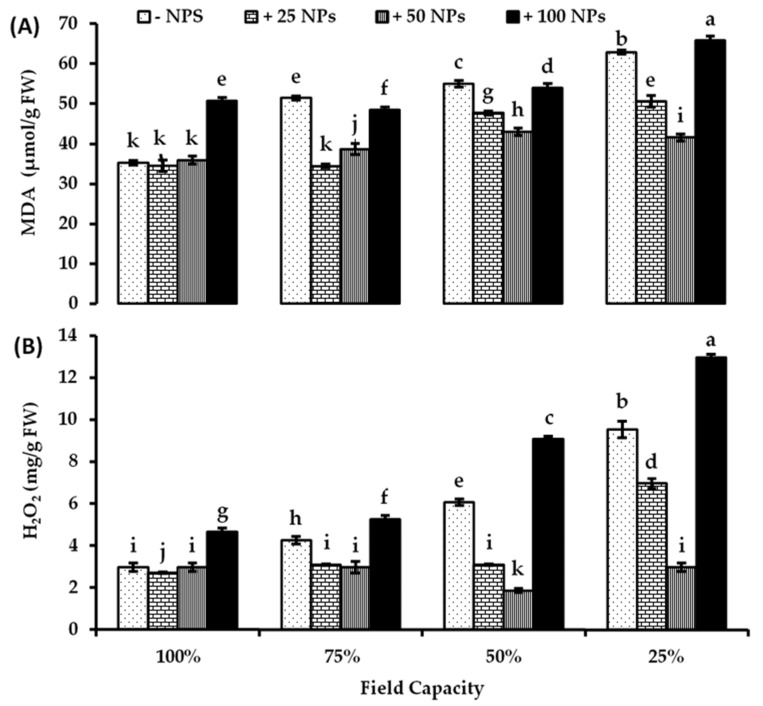
Stress indicators, (**A**) malondialdehyde (MDA) and (**B**) hydrogen peroxide (H_2_O_2_) concentration in tomato leaves as affected by foliar spraying with different concentrations of ZnO-NPs under different levels of drought stress. Each point represents a mean value of three replicates (*n* = 3) with vertical bars representing the standard error of the mean. Bars with different letters indicate a significant difference (*p* < 0.05) between Zn-O NPs treatments at all studied water levels as determined by two-way ANOVA and Bonferroni multiple comparison test..

Accumulation of lipid peroxides is mainly an indicator for ROS acting on the bio-membrane. The peroxidation of membrane lipid is one of the most damaging of oxidative stress caused by exposing plant cells to drought stress, as shown in Figure 2A. No major variations were observed in fully watered plants due to treatment with ZnO-NPs at lower concentrations (25 and 50 mg/L). However, compared with the untreated control, 100 mg/L ZnO-NPs significantly increased the content of MDA by about 43.92%, indicating that oxidative stress under 100% FC was caused by 100 mg/L ZnO-NPs. On the other hand, 25 and 50 mg/L ZnO-NPs significantly decreased MDA content lower than untreated controls under all investigated drought levels. ZnO-NPs (25 mg/L) reduced the MDA concentration by about 33.12, 13.29 and 19.50% less than controls under 75%, 50%, and 25% FC, respectively. ZnO-NPs (50 mg/L) reduced MDA concentration by about 24.82, 21.85, and 33.88% less than controls under 75, 50, and 25% FC, respectively. ZnO-NPs (100 mg/L) did not significantly affect the MDA concentration under all drought levels relative to their corresponding control (Figure 2A). There were significant effects of drought levels, ZnO-NPs treatments, and their interaction on MDA concentration in tomato leaves based on two-way ANOVA (Table S2). 

In accordance with MDA results, H_2_O_2_ concentration increased significantly by increasing water stress in tomato leaves (Figure 2B). No significant differences were observed in H_2_O_2_ content by sparing ZnO-NPs at lower concentrations (25 and 50 mg/L) in water unstressed plants. ZnO-NPs (25 and 50 mg/L) significantly reduced H_2_O_2_ content under all drought levels lower than their untreated controls. At 50 and 25% FC, 50 mg/L ZnO-NPs made a large difference to the H_2_O_2_ concentration by about 69.30 and 68.83%, respectively, of their untreated controls. Under all water levels, 100 mg/L ZnO-NPs significantly increased the H_2_O_2_ concentration compared to their corresponding controls, which in turn motivates oxidative stress under all water levels (Figure 2B). There were significant effects of drought levels, ZnO-NPs treatments, and their interaction on H_2_O_2_ concentration in tomato leaves based on two-way ANOVA (Table S2). 

**Figure 3:**
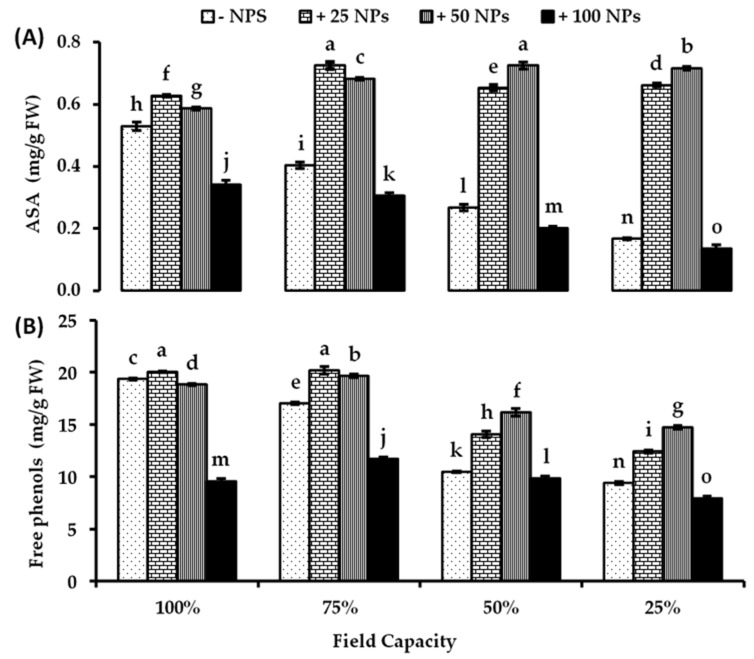
Antioxidants, (**A**) ascorbic acid (AsA), and (**B**) free phenols concentration in tomato leaves as affected by foliar spraying with different concentrations of ZnO-NPs under different levels of drought stress. Each point represents a mean value of three replicates (*n* = 3) with vertical bars representing the standard error of the mean. Bars with different letters indicate a significant difference (*p* < 0.05) between Zn-O NPs treatments at all studied water levels as determined by two-way ANOVA and Bonferroni multiple comparison test.

Increasing water stress significantly reduced the AsA concentration in tomato leaves reaching its lowest value at severe drought conditions (25% FC) (Figure 3A). Increased antioxidants mean, in turn, inhibition of oxidative stress. According to the results demonstrated in Figure 3A, application of lower ZnO-NPs concentrations (25 and 50 mg/L) significantly enhanced the AsA content in the tomato leaves both in water unstressed and stressed plants. By applying 25 mg/L ZnO-NPs, the AsA content increased by about 18.49, 79.46, 144.57, and 296.41% higher than untreated controls under all water levels of 100, 75, 50, and 25% FC, respectively. ZnO-NPs (50 mg/L) increased of AsA content by about 10.75, 69.06, 171.54, and 329.34% higher than untreated controls under water levels of 100, 75, 50, and 25% FC, respectively. In contrast, 100 mg/L ZnO-NPs lowered AsA content at all investigated water regimes (Figure 3A). There were significant effects on drought levels, ZnO-NPs treatments, and their interaction on AsA concentrations in tomato leaves based on two-way ANOVA (Table S3). 

The results in Figure 3B show that increasing drought conditions show a significant reduction in free phenols content. It was noticeable that foliar spray of 25 and 50 mg/L ZnO-NPs significantly increased the free phenols content under all water stress levels compared to their corresponding controls. At moderate and severe drought conditions (50 and 25% FC), 50 mg/L ZnO-NPs recorded the largest increase in the amount of free phenols by about 54.50 and 56.80% consecutively compared with their respective controls. Alternatively, 100 mg/L ZnO-NPs is associated with a higher reduction in free phenols content under all investigated water levels compared to ZnO-NPs untreated controls (Figure 3B). There were significant effects of drought levels, ZnO-NPs treatments, and their interaction on free phenols concentration in tomato leaves based on two-way ANOVA (Table S3). 

### 2.3. Antioxidant Enzymes

**Figure 4:**
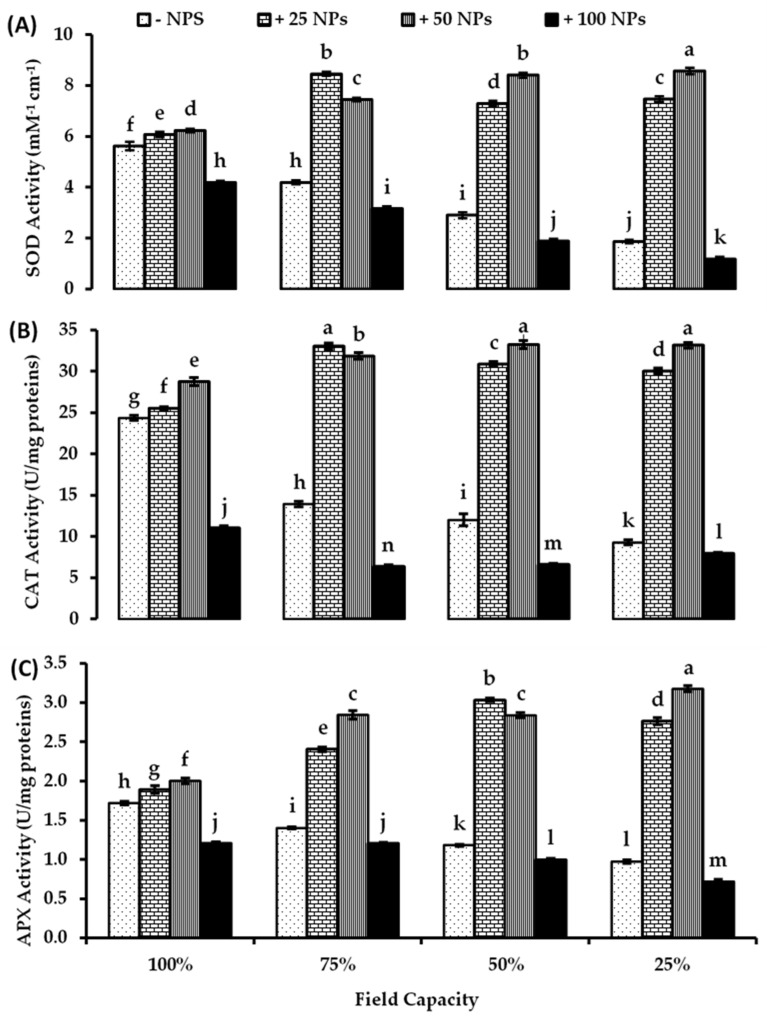
Antioxidant enzymes, (**A**) superoxide dismutase (SOD), (**B**) catalase (CAT), and (**C**) ascorbate peroxidase (APX) activity in tomato leaves as affected by foliar spraying with different concentrations of ZnO-NPs under different levels of drought stress. Each point represents a mean value of three replicates (*n* = 3) with vertical bars representing the standard error of the mean. Bars with different letters indicate a significant difference (*p* < 0.05) between Zn-O NPs treatments at all studied water levels as determined by two-way ANOVA and Bonferroni multiple comparison test.

As presented in Figure 4, the activity of all studied antioxidant enzymes in tomato leaves decreased significantly by increasing drought stress conditions. However, the foliar spraying with 25 and 50 mg/L ZnO-NPs positively affected the enzymes’ activity under all studied water regimes. At a mild water deficit level (75% FC), the data clarified that 25 mg/L ZnO-NPs enhanced SOD, CAT, and APX activity by about 101.83, 137.11, and 71.31% consecutively compared with their respective controls. Moreover, 50 mg/L ZnO-NPs enhanced SOD, CAT, and APX activity by about 77.70, 128.79, and 102.46%, respectively, higher than untreated control under mild drought conditions. At moderate stress conditions (50% FC), SOD, CAT, and APX activity were enhanced in response to 25 mg/L ZnO-NPs by about 148.70, 156.96, and 156.31% consecutively higher than their respective controls. Similarly, 50 mg/L ZnO-NPs enhanced SOD, CAT, and APX activity by about 186.67, 276.82, and 139.81% consecutively higher than the untreated control under 50% FC. At severe drought stress, 25 mg/L ZnO-NPs induced SOD, CAT, and APX activity by about 3.99-, 3.23-, and 2.82-fold of their corresponding control, respectively. Likewise, at 25% FC, SOD, CAT, and APX activity increased with 50 mg/L ZnO-NPs by about 4.58-, 3.57-, and 3.25-fold consecutively compared with their respective control. Nonetheless, a significant reduction in the activity of all studied enzymes was recorded due to the treatment with 100 mg/L ZnO under all studied water conditions (Figure 4A,B,C). There were significant effects of drought levels, ZnO-NPs treatments, and their interaction on the activity of all investigated antioxidant enzymes in tomato leaves based on two-way ANOVA (Table S4).

## 3. Discussion

Due to eco-friendliness, economic opportunities, and sustainability, the “green” route for NPs synthesis is of great interest. It is a new and evolving research area in the scientific world, where regular advances are noted to guarantee a promising future for this field that can be used to reduce the negative effects of abiotic stresses on plants [31]. Zinc is an important nutrient that plays an important role as a cofactor for different enzymes and as a major structural component of regulatory proteins, therefore, impacting many plant physiological processes [32]. Kabir et al. [33] reported that Zn deficiency negatively affected the biomass, cellular integrity, and chlorophyll synthesis in tomato. In this study, it was found that tomato biomass decreased steadily by an increasing drought level. However, foliar application of ZnO-NPs, at specific concentrations (25 and 50 mg/L), showed increased biomass output compared to ZnO-NPs untreated controls under all studied water levels. In accordance with our results, the foliar spray of ZnO-NPs boosted up all and growth parameters in wheat and peanuts plants [34,35].

Drought results in the accumulation of ROS, including H_2_O_2_, leading to cell membrane system lipid peroxidation, which is primarily due to the development of MDA [36,37]. In this sense, foliar spray of ZnO-NPs at the optimum concentrations reduced the accumulation of MDA and H_2_O_2_ in tomato leaves under drought conditions (75, 50, and 25% FC) compared to the untreated control. In this regard, our results are supported by Sun et al. [38], who concluded that ZnO-NPs treatment decreased the levels of H_2_O_2_ and MDA in maize leaves, minimizing drought-induced lipid peroxidation while preserving plasma membrane stabilization. The significant enhancement in stress indicators caused by 100 mg/L ZnO-NPs treatment in fully watered plants could be attributed to Zn phytotoxicity at higher concentrations as mentioned by Catalina et al. [39], who concluded that high Zn concentrations are potentially toxic to all organisms. The results of the current study also indicated that 100 mg/L ZnO-NPs significantly reduced tomato fresh and dry biomass under 100% FC. Zinc toxicity-associated clues in plants include reduced yield and stunted growth and reduced export of photoassimilates from leaves to roots [40]. Similarly, in wheat leaves, Zn excess caused a significant enhancement of H_2_O_2_ and MDA levels and reduced photosynthetic pigments concentrations [41]. Jain et al. [42], reported that application of 130 mg/L Zn reduced sugarcane growth and increased oxidative stress as indicated by increased H_2_O_2_ and MDA levels. Ascorbic acid protects cells from oxidative damage, leads to vitamin C regeneration, and serves as a cofactor for enzymes involved in flavonoid and phytohormone biosynthesis [43]. Therefore, plants with reduced levels of AsA are hypersensitive to stress conditions [44] which is supported by the results of our study, where AsA was markedly reduced by increasing drought stress conditions. Furthermore, in order to scavenge free radicals, phenolic compounds are also documented to play a vital role and their content is increased under different abiotic stresses. Increased concentrations of AsA and phenolic compounds in tomato leaves have been reported in the present study when 25 and 50 mg/L ZnO-NPs were applied under all drought levels. These findings are consistent with those previously reported by Iziy et al. [45], in which exogenous application of ZnO-NPs improved the phenolic content in *Portulaca oleracea* L. The significant reduction in AsA and phenols concentration and induction of MDA and H_2_O_2_ in response to foliar application of 100 mg/L ZnO-NPs indicates decreases in antioxidant potential which could be due to Zn toxicity, since Zn, as a heavy metal, at this concentration, could be too high for tomato plants. Javed et al. [46] studied the effect of ZnO-NPs on the phenolic content of *Stevia rebaudiana* and showed a marked decrease in phenols content due to treatment with 100 and 1000 mg/L NPs which results in a decrease in antioxidant activity.

The activity of the studied antioxidant enzymes (SOD, CAT, and APX) was enhanced by applying 25 and 50 mg/L ZnO-NPs. Nevertheless, water deficiency had a negative impact on the enzyme activities in untreated plants, likewise to plants that were sprayed with 100 mg/L ZnO-NPs. Our outcomes are similar to prior studies that documented an increased CAT activity by ZnO-NPs treatments in leaves, while decreased APX activity at concentrations greater than 500 mg/L [47]. Exposure to ZnO-NPs in *Gossypium hirsutum* resulted in increased activities of SOD and CAT, with a consequent reduction in lipid peroxidation after treatment with ZnO-NPs [48]. The improved antioxidant enzymes activity mediated by ZnO-NPs application in tomato was also recorded under salt stress conditions by Faizan et al. [24]. In the same context, the relative transcript abundance of Cu/Zn-SOD, APX, and CAT were significantly up-regulated in ZnO-NPs-treated maize and wheat under drought conditions [38,49]. Thus, the NPs were probably helped in scavenging ROS by activating antioxidant enzyme systems [50]. Significantly higher antioxidant enzymes activities in ZnO-NPs-treated plants reduced the H_2_O_2_ and MDA levels in maize leaves, which reduced the drought-induced lipid peroxidation, hence maintaining the stabilization of chloroplasts and mitochondria [51]. Superoxide dismutase catalyzes superoxide free radical dismutation into H_2_O_2_ and O_2_ which are recognized as early defenses against free radicals of oxygen in the cytosol, chloroplast, and mitochondria [52]. It is the key antioxidant enzyme protector against oxidative stress triggered by ROS, which is a major scavenger of O_2_^·−^ free radicals converted into H_2_O and O_2_ by catalase and a variety of peroxidases [53]. CAT can efficiently break down high concentrations of H_2_O_2_ and reduce the damage of ·OH produced by H_2_O_2_. Therefore, the level of hydrogen peroxide is controlled by CAT in plant cells. APX is another enzyme involved in the removal of H_2_O_2_. It should be noted that this enzyme participates in controlling slight changes in H_2_O_2_ concentration [54]. These three key antioxidant enzymes acted synergistically and scavenged the ROS efficiently in vivo. Overall, the current study proved that, in response to green ZnO-NPs foliar application, non-enzymatic antioxidants such as AsA and phenolic compounds work in a coordinated fashion with antioxidant enzymes such as SOD, CAT, and APX, in tomato plants to reduce the negative effects of drought stress by reducing oxidative stress. 

## 4. Materials and Methods

### 4.1. Experimental Design and ZnO-NPs Treatments

This experiment was conducted at the experimental station at King Abdulaziz University in the Kingdom of Saudi Arabia during the autumn season, starting from the 1st of October until the 26th of November of 2020. The weather during this period was slightly windy with a gradual drop in temperature and moderate humidity. The average daily minimum temperature was 24 °C at night and in the early morning, while the average daily maximum temperature inside the greenhouse was 32 °C during the day. Tomato (*Lycopersicum esculentum* Mill) seeds were planted in plastic containers filled with 3 kg of homogeneously mixed sand: clay soil (2:1). The plants were cultivated under natural light conditions in the greenhouse and were irrigated with tap water at field capacity (FC) regularly every two days. The FC of the used soil mixture was determined by the gravimetric method following the method described by Souza et al. [55], which consists of the difference between the wet soil after saturation and free drainage and the weight of the dry soil. The pots were thinned after germination to 5 seedlings per pot. The drought stress was applied after the appearance of the fourth leaf (30-day-old seedling) at four levels, control (100% FC), mild stress (75% FC), moderate stress (50% FC), and severe stress (25% FC) based on the managed allowed depletion [56]. The soil moisture level of the pots was controlled every 2 days by weighing the pots and the amount of water lost through soil evaporation, and transpiration was replaced for water level. ZnO-NPs (25, 50, 100 mg/L) were sprayed in two stages: at the beginning of drought stress and seven days after drought stress application. For control plants (0 mg/L ZnO-NPs), the same volume of distilled water was sprayed. After two weeks, regular watering with 100% FC was resumed for all pots after the water stress period, to allow the plants to recover for one week. Then, tomato seedlings (51-day-old) were harvested for biomass determination and biochemical analysis. Fresh leaves samples were stored in the freezer (−80 °C) for measuring biochemical traits. The experiment was carried out with a complete random design in three replicates.

### 4.2. Synthesis of ZnO-NPs Using C. forskohlii Leaves Extract

**Figure 5:**
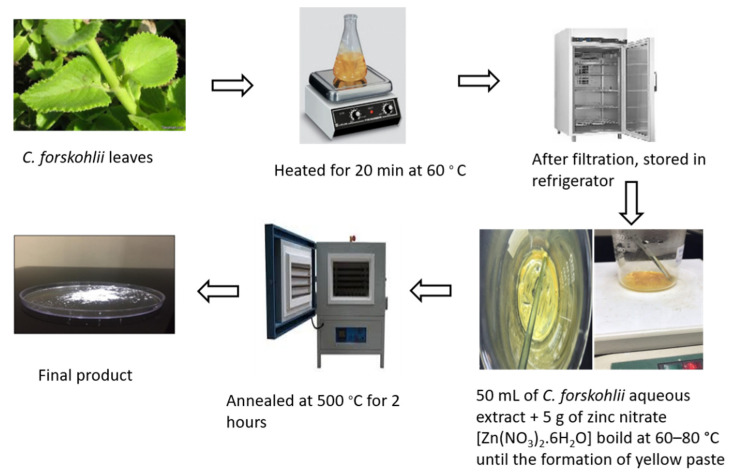
Flow chart for synthesis of zinc oxide nanoparticles.

**Figure 6:**
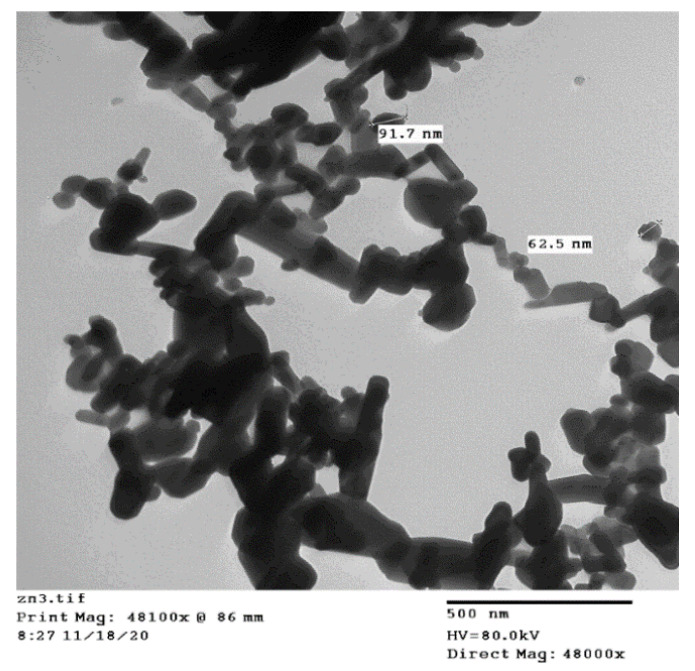
TEM image of synthesized zinc oxide nanoparticles.

*Coleus forskohlii* Briq. is a common indigenous medicinal plant belonging to the family Lamiaceae. In addition to the therapeutic compounds, many other actives phytochemicals extracted from *C. forskohlii*. show various biological activities [57]. The chemical composition of its extract is reported by Kanne et al. [58]. Therefore, *C. forskohlii* leaves were used as a bio-reducing agent in this study for ZnO-NPs preparation. Fresh leaves (10 g) were washed thoroughly under running tap water followed by double-distilled water and then cut and soaked in a 250 mL flask containing 100 mL double-distilled water. The solution was kept at 60 °C on a hot plate for 20 min until the color of the aqueous solution became yellowish. After that, the yellow-colored extract was cooled to room temperature and filtered with Whatman no.1 filter paper, then stored in the refrigerator to use for ZnO-NPs synthesis. For the synthesis of ZnO-NPs, 50 mL of *C. forskohlii* aqueous extract was taken from the stock solution (stored in the refrigerator). Then, 5 g of zinc nitrate [Zn(NO_3_)_2_·6H_2_O] was dissolved in the *C. forskohlii* extract solution under constant stirring using a magnetic stirrer. After complete dissolution of the mixture, the solution was boiled at 60–80 °C by using a magnetic stirrer until the formation of a deep yellow colored paste. The paste was transferred to a ceramic crucible cup and heated in a furnace at 500 °C for 2 h. The obtained white-colored powder was used for further studies. The flowchart used for the preparation of ZnO-NPs is shown in Figure 5. Before use in tomato treatment, the synthesized ZnO-NPs were characterized by UV-Visible spectroscopy [UV-1800], transmission electron microscopy (TEM) [Mic JEM 1011], Fourier transform infrared (FTIR) [NICOLET iN10], and X-ray diffraction (XRD) [59]. Figure 6 shows the TEM image of the synthesized ZnO-NPs.

### 4.3. Plant Biomass Determination 

The samples were washed with distilled water and gently dried with tissue paper. The freshly harvested plants were separated into shoots and roots and weighted for fresh biomass determination. Then, the samples were wrapped in foil paper and oven-dried at 70 °C until a constant weight of each sample was reached (approximately after 48 h), to assess the dry weight.

### 4.4. Malondialdehyde Determination

The degree of lipid peroxidation was calculated by measuring the malondialdehyde (MDA) formation with thiobarbituric acid (TBA) using the procedure by Narwal et al. [60]. In short, fresh leaf tissue samples (0.1 g) were homogenized with 1.5 mL trichloroacetic acid (TCA) (0.1%). At 10,000 rpm for 10 min, the resulting homogenate was centrifuged, and 1 mL of the supernatant was added to 2 mL of 20% TCA containing 0.5% TBA. The extract was heated for 30 min in a water bath at 95 °C and then quickly cooled in an ice bath. After that, the extract was centrifuged at 10,000 rpm for 10 min. The supernatant absorbance was read at 532 and 600 nm against a blank using a UV/Vis spectrophotometer (Perkin Elmer, Lambda 25)**.**

### 4.5. Hydrogen Peroxide Determination 

Hydrogen peroxide content in tomato leaves was determined using a modified method by Mukherjee and Choudhuri [61]. Leaf samples (0.1 g) were extracted in cold acetone. An aliquot of 3 mL of the extract was mixed with 5 mL of titanium dioxide reagent. The strength of the developed yellow color was estimated at 415 nm using a UV-Vis spectrophotometer.

### 4.6. Ascorbic Acid Determination 

According to Mukherjee and Choudhuri [61], the ascorbic acid concentration was determined. Leaf tissues (0.2 g) were ground with liquid N2 and suspended in 2 mL of 5% TCA. At 10,000 rpm, the homogenate was centrifuged at 5 °C for 15 min. Then, 0.8 mL of 10% TCA was added to 0.2 mL of tissue homogenate. After shaking, the tubes were placed in an ice bath for 5 min and then centrifuged for another 5 min at 3000 rpm. Next, 0.5 mL of the extract was diluted to 2.0 mL using distilled water, then, 0.2 mL of diluted Folin-Ciocalteu’s reagent was added to the extract and the tubes were vigorously shaken. The absorption of the produced blue color was measured after 10 min at 760 nm against a blank using a UV-Vis spectrophotometer

### 4.7. Free phenols Determination 

According to Kofalvi and Nassuth [62], free phenolic compounds were determined. Leaf samples (0.5 g) were extracted for 90 min at 80 °C in 50% methanol (1:2 *v*/*v*) The extract was then centrifuged for 15 min at 14,000 rpm. Using the Folin-Ciocalteu’s assay, the supernatant was assessed for free phenols determination. One hundred microliters of the extract was diluted with water to 1 mL and mixed with 0.5 mL of 2 N Folin and 2.5 mL of 20% Na_2_CO_3_ reagents. After 20 min, at room temperature, the absorbance of the samples was measured at 725 nm.

### 4.8. Antioxidant Enzymes Activity

Enzymes extract was prepared according to Cakmak and Marschner [63]. Leaf tissue (0.5 g) was ground in liquid N_2_ to a fine powder. After that, the leaf tissue was suspended in 5 mL of 100 mM potassium phosphate buffer (pH 7.8) containing 0.1 mM ethylenediamine tetraacetic acid (ETDA) and 0.1 g polyvinylpyrrolidone. The homogenate was centrifuged at 18,000 rpm for 10 min at 4 °C, and the supernatants were collected and used for enzymes activity assays.

#### 4.8.1. Superoxide Dismutase (SOD, EC 1.15.1.1)

Superoxide dismutase activity was calculated by epinephrine autoxidation, as indicated by Cakmak and Marschner [63]. 

A final volume of 2 mL of the reaction medium containing 25 mM of sodium carbonate buffer (pH 10.2), 200 μL of 0.5 mM of EDTA, and 100 μL of enzyme extract was assessed for enzyme activity. The reaction was initiated by adding 100 μL of 15 mM epinephrine (dissolved in 10 mM HCl, pH 2.4). At 480 nm, autoxidation of epinephrine was determined using a UV-Vis spectrophotometer.

#### 4.8.2. Catalase (CAT, EC 1.11.1.6)

Catalase activity was spectrophotometrically assayed by monitoring the absorbance shift at 240 nm due to the reduced absorption of H_2_O_2_ [64]. The reaction medium contained 2.4 mL of 50 mM of potassium phosphate buffer (pH 7) and 500 μL of enzyme extract. The reaction was initiated by adding 100 μL of H_2_O_2_ (10 mM). 

#### 4.8.3. Ascorbate Peroxidase (APX, EC 1.11.1.11)

The performance of ascorbate peroxidase was calculated by the method proposed by Zhang and Kirkham [64]. In a reaction mixture containing 50 mM potassium phosphate buffer (pH 7), 5 mM H_2_O_2_, 0.1 mM Na_2_-ETDA, 0.5 mM AsA, and 50 μL enzyme extract, the rate of H_2_O_2_-dependent AsA oxidation was determined using a UV-Vis spectrophotometer.

### 4.9. Statistical Analysis

The data were analyzed using the statistical software SPSS version 21.0. A two-way analysis of variance (ANOVA) was performed to examine the effects of the studied water levels, ZnO-NPs treatments, and their interactions upon all investigated traits. Significant differences between treatments (*p* < 0.05) were confirmed using Bonferroni multiple comparison test. All values were expressed with their standard error (SE) as a mean value of three replicates.

## 5. Conclusions

This study provides important evidence about the effect of green ZnO-NPs in alleviating drought stress in tomato plants. Foliar spraying ZnO-NPs biosynthesized using *Coleus forskohlii* leaf extract promotes tomato growth and the antioxidant system under all tested drought levels. Out of the investigated concentrations (25, 50, and 100 mg/L), 25 and 50 mg/L ZnO-NPs increased the shoot and root biomass, reduced stress indicators, and enhanced the antioxidant system of tomato plants more significantly. The highest concentration tested (100 mg/L ZnO-NPs) enhanced the oxidative stress both in water-stressed and unstressed plants, and therefore, should be considered as a phytotoxic concentration. Therefore, it can be concluded that foliar application of green ZnO-NPs at concentrations of 25 and 50 mg/L ZnO-NPs is recommended for the enhancement of drought tolerance in tomato plants.

## Data Availability

The data presented in this study are available on request from the corresponding author.

## Supplementary Materials

The following are available online at https://www.mdpi.com/article/10.3390/plants10112400/s1, Table S1. Tests of between-subject’s effects (Two-Way ANOVA) on shoot snd root FW and DW; Table S2. Tests of between-subject’s effects (Two-Way ANOVA) on stress indicators; Table S3. Tests of between-subject’s effects (Two-Way ANOVA) on antioxidants; Table S4. Tests of between-subject’s effects (Two-Way ANOVA) on antioxidant enzymes.
