# Supplementary material for: Foliar Sprayed Green Zinc Oxide Nanoparticles Mitigate Drought-Induced Oxidative Stress in Tomato
Source: Plants (Basel). 2021 Nov 7;10(11):2400. doi: 10.3390/plants10112400 (PMC8622210; doi:10.3390/plants10112400)
Supplement: Supplementary file 1 [file plants-10-02400-s001.zip › plants-1436528-supplementary.pdf]

Table S1: Tests of Between-Subjects Effects (Two-Way ANOVA) on Shoot and Root FW and DW

Source	Type III Sum of Squares	df	Mean Square	F	Sig.
**Shoot FW**					
Corrected Model	149.120[a]	15	9.941	80.072	.000
Intercept	4183.760	1	4183.760	33697.700	.000
Water levels	48.995	3	16.332	131.541	.000
ZnO-NPs	62.288	3	20.763	167.230	.000
Water levels * ZnO-NPs	37.838	9	4.204	33.862	.000
Error	3.973	32	.124		
Total	4336.854	48			
Corrected Total	153.093	47			
**Shoot DW**					
Corrected Model	31.484[a]	15	2.099	3727.603	.000
Intercept	154.998	1	154.998	275267.316	.000
Water levels	.843	3	.281	499.114	.000
ZnO-NPs	24.833	3	8.278	14700.417	.000
Water levels * ZnO-NPs	5.808	9	.645	1146.161	.000
Error	.018	32	.001		
Total	186.501	48			
Corrected Total	31.502	47			
**Root FW**					
Corrected Model	16.071[a]	15	1.071	2999.734	.000
Intercept	15.148	1	15.148	42411.994	.000
Water levels	1.393	3	.464	1299.919	.000
ZnO-NPs	10.530	3	3.510	9827.617	.000
Water levels * ZnO-NPs	4.148	9	.461	1290.378	.000
Error	.011	32	.000		
Total	31.231	48			
Corrected Total	16.083	47			
**Root DW**					
Corrected Model	11.557[a]	15	.770	67364.548	.000
Intercept	2.254	1	2.254	197050.639	.000
Water levels	2.436	3	.812	70990.365	.000
ZnO-NPs	2.317	3	.772	67517.457	.000
Water levels * ZnO-NPs	6.805	9	.756	66104.973	.000
Error	.000	32	1.144E-5		
Total	13.811	48			
Corrected Total	11.558	47			

Table S2:Tests of Between-Subjects Effects (Two-Way ANOVA) on Stress Indicators

MDA

Source	Type III Sum of Squares	df	Mean Square	F	Sig.
Corrected Model	4374.181[a]	15	291.612	116.116	.000
Intercept	105492.340	1	105492.340	42005.703	.000
Water levels	1827.889	3	609.296	242.614	.000
ZnO-NPs	1873.745	3	624.582	248.700	.000
Water levels * ZnO-NPs	672.547	9	74.727	29.756	.000
Error	80.364	32	2.511		
Total	109946.885	48			
Corrected Total	4454.545	47			

H2O2

Source	Type III Sum of Squares	df	Mean Square	F	Sig.
Corrected Model	434.529[a]	15	28.969	278.658	.000
Intercept	1242.805	1	1242.805	11954.930	.000
Water levels	163.568	3	54.523	524.470	.000
ZnO-NPs	189.196	3	63.065	606.646	.000
Water levels * ZnO-NPs	81.764	9	9.085	87.391	.000
Error	3.327	32	.104		
Total	1680.661	48			
Corrected Total	437.855	47			

[a]. R Squared = .999 (Adjusted R Squared = .999)

Table S3: Tests of Between-Subjects Effects (Two-Way ANOVA) on Antioxidants

Source	Type III Sum of Squares	df	Mean Square	F	Sig.
**ASA**					
Corrected Model	2.133[a]	15	.142	602.277	.000
Intercept	11.207	1	11.207	47460.088	.000
Water levels	.096	3	.032	135.297	.000
ZnO-NPs	1.775	3	.592	2505.654	.000
Water levels * ZnO-NPs	.262	9	.029	123.479	.000
Error	.008	32	.000		
Total	13.348	48			
Corrected Total	2.141	47			
**Free Phenols**					
Corrected Model	853.512[a]	15	56.901	402.423	.000
Intercept	10004.031	1	10004.031	70752.167	.000
Water levels	341.951	3	113.984	806.133	.000
ZnO-NPs	418.541	3	139.514	986.692	.000
Water levels * ZnO-NPs	93.021	9	10.336	73.097	.000
Error	4.525	32	.141		
Total	10862.068	48			
Corrected Total	858.037	47			

[a]. R Squared = .999 (Adjusted R Squared = .999)

Table S4: Tests of Between-Subjects Effects (Two-Way ANOVA) on Antioxidant Enzymes

SOD

Source	Type III Sum of Squares	df	Mean Square	F	Sig.
Corrected Model	4.083[a]	15	.272	4360.448	.000
Intercept	18.710	1	18.710	299704.807	.000
Water levels	.104	3	.035	553.948	.000
ZnO-NPs	3.272	3	1.091	17471.503	.000
Water levels * ZnO-NPs	.707	9	.079	1258.930	.000
Error	.002	32	6.243E-5		
Total	22.795	48			
Corrected Total	4.085	47			

CAT

Source	Type III Sum of Squares	df	Mean Square	F	Sig.
Corrected Model	59.619[a]	15	3.975	2808.574	.000
Intercept	237.062	1	237.062	167515.055	.000
Water levels	.395	3	.132	93.094	.000
ZnO-NPs	53.400	3	17.800	12577.923	.000
Water levels * ZnO-NPs	5.824	9	.647	457.284	.000
Error	.045	32	.001		
Total	296.726	48			
Corrected Total	59.664	47			

APX

Source	Type III Sum of Squares	df	Mean Square	F	Sig.
Corrected Model	.239[a]	15	.016	607.337	.000
Intercept	1.306	1	1.306	49839.364	.000
Water levels	.005	3	.002	63.436	.000
ZnO-NPs	.194	3	.065	2473.047	.000
Water levels * ZnO-NPs	.039	9	.004	166.734	.000
Error	.001	32	2.621E-5		
Total	1.546	48			
Corrected Model	.239[a]	15	.016	607.337	.000

[a]. R Squared = .999 (Adjusted R Squared = .999)
